# Shear Bond Strength of Artificial Teeth to Conventional and Computer-Aided Design/Computer-Aided Manufacturing (CAD/CAM) Denture Bases: An In Vitro Comparison

**DOI:** 10.7759/cureus.98562

**Published:** 2025-12-06

**Authors:** Subhra Rout, Reena Mittal, Ravi Madan, Vinay Rana, Prachi Madan, Mouli Sardar, Krithika J, Meenakshi Tyagi, Seema Gupta

**Affiliations:** 1 Department of Prosthodontics, Kothiwal Dental College and Research Centre, Moradabad, IND; 2 Department of Orthodontics, Kothiwal Dental College and Research Centre, Moradabad, IND

**Keywords:** acrylic composite, artificial teeth, computer-aided design, computer-aided manufacturing, polymethyl methacrylate

## Abstract

Introduction: Complete dentures remain essential for restoring function and aesthetics in edentulous patients; however, tooth debonding continues to be a leading cause of repair. This in vitro study aimed to compare the shear bond strengths of three types of artificial teeth (crosslinked acrylic, composite, and computer-aided design/computer-aided manufacturing as CAD/CAM-milled teeth) bonded to conventional heat-cured polymethyl methacrylate (PMMA) and CAD/CAM-milled denture base resins.

Materials and methods: Sixty cylindrical specimens (20 mm in height × 25 mm in diameter) were divided into two main groups (n = 30 each) based on the denture base material: conventional heat-cured PMMA and CAD/CAM-milled PMMA. Each group was subdivided (n = 10) according to tooth type: crosslinked acrylic, composite, and CAD/CAM-milled teeth. Teeth were bonded following the manufacturer's protocols for the CAD/CAM group and the standard packing technique for the heat-cured group. The shear bond strength was measured using a universal testing machine at a crosshead speed of 1 mm/min. Data were analyzed using one-way and two-way analyses of variance with post-hoc tests (p < 0.05).

Results: Mean shear bond strength values were as follows: conventional heat-cured base with composite teeth (272.11 ± 85.44 MPa), acrylic teeth (179.09 ± 74.71 MPa), and CAD/CAM-milled teeth (174.47 ± 43.13 MPa); CAD/CAM-milled denture base with CAD/CAM-milled teeth (359.56 ± 76.62 MPa), acrylic teeth (275.51 ± 89.69 MPa), and composite teeth (237.23 ± 75.71 MPa). Two-way analysis of variance revealed significant main effects for both denture base material and tooth type, with a highly significant interaction effect (p < 0.001, η²p = 0.28). The strongest bond was achieved with CAD/CAM-milled teeth on CAD/CAM-milled denture bases, which was significantly higher than that of all other combinations.

Conclusion: Bond strength is highly dependent on material compatibility. The combination of CAD/CAM-milled teeth and bases from pre-polymerized high-pressure PMMA provides superior adhesion. In conventional heat-cured systems, the composite teeth yield the strongest bond. For optimal clinical durability in digital workflows, matched CAD/CAM tooth-based systems should be preferred.

## Introduction

Complete dentures remain a cornerstone in the rehabilitation of edentulous patients and offer a cost-effective and biocompatible solution to restore aesthetics, function, and phonetics. Since its introduction, polymethyl methacrylate (PMMA) has been the material of choice for denture bases because of its ease of fabrication, biocompatibility with oral tissues, aesthetic appeal, repairability, and affordability [1]. However, traditional heat-cured PMMA dentures are prone to polymerization shrinkage, dimensional instability, and residual monomer release, which can lead to deformations, loss of retention, allergic reactions, and mucosal irritation [1,2]. The conventional fabrication process is labor-intensive and requires multiple clinical appointments, typically five or more, for impressions, jaw relations, try-ins, and processing, often spanning weeks or months. These limitations can compromise fit, masticatory efficiency, and overall patient satisfaction [1].

Advancements in digital dentistry have revolutionized denture fabrication using computer-aided design and computer-aided manufacturing (CAD/CAM) techniques. Introduced more than three decades ago, CAD/CAM systems enable precise digital impressions, virtual design, and milling or additive manufacturing of dentures, reducing chair time, laboratory steps, and errors associated with manual processes [3]. CAD/CAM denture bases milled from pre-polymerized PMMA discs under high temperature and pressure exhibit superior mechanical properties, including higher elastic modulus, toughness, and dimensional stability, with lower residual monomer content than conventional heat-cured resins [4]. These enhancements minimize polymerization shrinkage and improve clinical outcomes such as better retention and reduced tooth movement [5]. Despite these benefits, the integration of artificial teeth poses unique challenges in CAD/CAM workflows, particularly regarding the bond strength to the denture base.

Tooth debonding is a prevalent clinical issue, accounting for up to 22-30% of denture repairs, often owing to incompatible materials, surface contamination, or inadequate bonding mechanisms [6,7]. Various prefabricated teeth, including crosslinked acrylic, composite, and CAD/CAM-milled types, influence bond integrity differently [7]. While mechanical and chemical surface treatments (such as monomer application and sandblasting) have been explored to enhance adhesion, inconsistencies in the reported shear bond strengths arise from non-standardized testing and material variations [8]. Studies have indicated that CAD/CAM resins may offer comparable or superior bonding, but comprehensive comparisons with heat-cured resins across tooth types are limited [9,10].

This in vitro study aimed to evaluate and compare the shear bond strength between three different types of artificial teeth, namely crosslinked acrylic, composite, and CAD/CAM-milled, and two denture base resins, heat-cured PMMA and CAD/CAM-milled PMMA. The objectives were to assess the shear bond strength of each tooth type using both resins, compare these strengths across combinations, and identify the optimal pairing for enhanced durability and reduced debonding risk, thereby guiding clinical material selection and fabrication protocols.

## Materials and methods

Study design, setting, and eligibility

The present study was designed as an in-vitro comparative study and was conducted in the Department of Prosthodontics, Kothiwal Dental College and Research Centre, Moradabad, Uttar Pradesh, India. All laboratory procedures were performed under controlled environmental conditions to eliminate extraneous factors that could influence bonding outcomes. The entire research, including sample fabrication, testing, and data analysis, was completed over a period of 12 months, from January 2024 to December 2024. As this was a purely in vitro laboratory-based study involving no human or animal participants, informed consent and ethical approval were not applicable. All chemical waste materials were disposed of in accordance with the institutional biosafety and environmental protocols. Sample preparation for heat-cure groups adhered to the American Dental Association (ADA) Specification No. 182 (2021), whereas the CAD/CAM groups followed the International Organization for Standardization (ISO/TS 19736:2017) guidelines for bonding tests between polymer teeth and denture base materials.

Sample size estimation and grouping

The sample size was calculated using G*Power software (version 3.1.9.2, Heinrich-Heine-Universität Düsseldorf, Germany) with an effect size (Cohen’s d) of 0.50, an alpha error probability of 0.05, and a power of 80%. An effect size of 0.6 was derived from a previous study that evaluated the shear bond strength of various artificial teeth bonded to heat-cured denture base resin [11]. A total of 60 specimens were required to detect a statistically significant difference (30 specimens per main group). Accordingly, each of the six subgroups consisted of ten specimens.

Sixty standardized cylindrical specimens measuring 20 mm in height and 25 mm in diameter were fabricated for this study. These specimens were equally divided into two main groups of 30 each, based on the type of denture base resin used. Group 1 comprised specimens made with conventional heat-cure denture base resin (DPI Heat Cure; Dental Products of India, Mumbai, India), whereas Group 2 consisted of specimens prepared using CAD/CAM milled denture base resin from pre-polymerized pink PMMA discs (IvoBase CAD; Ivoclar Vivadent AG, Schaan, Liechtenstein).

Each main group was further subdivided into three subgroups of ten specimens each, according to the type of artificial tooth bonded to the resin cylinder. Subgroups 1A and 2A received cross-linked acrylic teeth (Ivostar/Gnathostar; Ivoclar Vivadent AG, Schaan, Liechtenstein), Subgroups 1B and 2B received prefabricated composite teeth (Dovis; Davis Schottlander & Davis Ltd., Letchworth, Hertfordshire, United Kingdom), and Subgroups 1C and 2C received CAD/CAM-milled artificial teeth fabricated from pre-polymerized white PMMA discs (IvoBase CAD; Ivoclar Vivadent AG, Schaan, Liechtenstein).

Fabrication of CAD/CAM-milled teeth

A maxillary central incisor of identical dimensions was scanned using an extraoral laboratory scanner (Exocad DentalCAD; exocad GmbH, Darmstadt, Germany). The resulting standard tesselation language (STL) file was imported into the CAD software, and 20 teeth were milled using a 5-axis milling machine (PrograMill PM7; Ivoclar Digital, Schaan, Liechtenstein).

Fabrication of Group 1 specimens (conventional heat-cured denture base)

Standardized wax cylinders (20 mm height × 25 mm diameter) with attached teeth were placed in Hanau flasks using Type III dental stone (Kalabhai Kalstone; Kalabhai Karson Pvt. Ltd., Mumbai, India). After dewaxing and application of a cold mold seal (DPI Cold Mould Seal; Dental Products of India), heat-cured acrylic resin (DPI Heat Cure) was mixed, packed in the dough stage, and processed using a short curing cycle (74 °C for 90 minutes, followed by terminal boiling at 100 °C for 30 minutes) in an acrylizer unit. The specimens were retrieved, finished, and polished to their final dimensions.

Fabrication of Group 2 specimens (CAD/CAM-milled denture base)

Wax cylinders of identical dimensions were scanned (Exocad scanner) and converted to STL format, and pink CAD discs were milled (PrograMill PM7). The ridge-lap surfaces of all the teeth were treated with methyl methacrylate monomers for 30 s. The teeth were then bonded to the milled bases using an IvoBase CAD Bond Kit (Ivoclar Vivadent AG, Schaan, Liechtenstein) strictly according to the manufacturer’s instructions under controlled pressure and light polymerization.

Shear bond strength testing

Each specimen was secured in a custom jig on a universal testing machine (Instron 8874; Instron Corporation, Canton, MA, USA). A flat shear blade was positioned parallel to the bonded interface on the palatal surface 1 mm above the ridge lap, and a load was applied at a crosshead speed of 1 mm/min until failure. The shear bond strength in megapascals (MPa) was calculated using the following formula: 

\[ \text{Shear bond strength (MPa)} = \frac{\text{Failure load (N)}}{\text{Bonded surface area (mm}^2\text{)}} \]

Statistical analysis

Data were recorded and analyzed using statistical software (IBM SPSS Statistics for Windows, version 25.0 (released 2017, IBM Corp., Armonk, NY)). Shear bond strength (MPa) values for all subgroups were recorded using a Microsoft Excel spreadsheet. The normality of the data was assessed using the Shapiro-Wilk test. Within each denture base-material group, differences in bond strength among the three subgroups were evaluated using one-way analysis of variance (ANOVA), followed by post-hoc Tukey’s test for pairwise comparisons. Between-group comparisons (across the two denture base materials and three tooth subgroups) were performed using two-way ANOVA, incorporating the interaction effect between denture base material and tooth subgroup, with Bonferroni post-hoc correction applied for multiple comparisons. Differences were considered statistically significant at p < 0.05, with adjustments made for multiple comparisons, where applicable.

## Results

One-way ANOVA revealed statistically significant differences in shear bond strength among the different artificial tooth material types within both denture base-material groups (p < 0.05). For the conventional heat-cured denture base group, the mean bond strength was highest for composite teeth (272.11 ± 85.44 MPa), followed by acrylic teeth (179.09 ± 74.71 MPa) and CAD/CAM-milled teeth (174.47 ± 43.13 MPa). A similar significant pattern was observed in the CAD/CAM-milled denture base group, where the CAD/CAM-milled teeth demonstrated the highest mean bond strength (359.56 ± 76.62 MPa). Acrylic teeth (275.51 ± 89.69 MPa) and composite teeth (237.23 ± 75.71 MPa) showed comparatively lower values (Table 1).

**Table 1:** Descriptive statistics and one-way analysis of variance (ANOVA) comparison of shear bond strength (MPa) among artificial tooth types within each denture base group. PMMA: polymethyl methacrylate, CAD/CAM: computer-aided design/computer-aided manufacturing, MPa: megapascals *p < 0.05 denotes statistical significance using one-way analysis of variance (ANOVA). Number of samples are presented as frequency (N) and percentage, whereas data for shear bond strength are presented as mean ± standard deviation (SD).

Groups	Subgroups	N (%)	95% Confidence interval for mean	Mean ± SD	F stats	p-value
Conventional heat-cured PMMA	Acrylic teeth	10 (16.67%)	125.65-232.53	179.09 ± 74.71	6.18	0.006*
	Composite teeth	10 (16.67%)	210.98-333.23	272.11 ± 85.44		
	CAD/CAM-milled teeth	10 (16.67%)	143.62-205.32	174.47 ± 43.13		
CAD/CAM-milled PMMA	Acrylic teeth	10 (16.67%)	211.35-339.67	275.51 ± 89.69	5.98	0.007*
	Composite teeth	10 (16.67%)	183.07-291.39	237.23 ± 75.71		
	CAD/CAM-milled teeth	10 (16.67%)	304.75-414.37	359.56 ± 76.62		

In the conventional heat-cured denture base group, a statistically significant difference was observed specifically between composite teeth and both acrylic teeth (p = 0.019) and CAD/CAM-milled teeth (p = 0.013), with composite teeth demonstrating a higher mean bond strength. No significant difference was found between the acrylic and CAD/CAM milled teeth (p = 1.000). For the CAD/CAM-milled denture base group, the only significant pairwise difference was between the composite and CAD/CAM-milled teeth (p = 0.007), where CAD/CAM-milled teeth had a significantly higher bond strength (Table 2).

**Table 2:** Tukey post-hoc pairwise comparisons of shear bond strength (MPa) among artificial tooth types within each denture base group. PMMA: polymethyl methacrylate, CAD/CAM: computer-aided design/computer-aided manufacturing, CI: confidence interval, MPa: megapascals *p < 0.05 denotes statistical significance using post-hoc Tukey’s test. Positive mean difference indicates the first subgroup has higher bond strength.

Groups	Pairwise comparison of subgroups	Mean difference (MPa)	T stats	p-value	95% CI lower limit	95% CI upper limit
Conventional heat-cured PMMA	Acrylic teeth vs. Composite teeth	-93.02	-2.97	0.019*	173.03	-13.00
	Acrylic teeth vs. CAD/CAM-milled teeth	4.62	0.15	1.000	-75.40	84.64
	Composite teeth vs. CAD/CAM-milled teeth	97.64	3.11	0.013*	17.62	177.66
CAD/CAM-milled PMMA	Acrylic teeth vs. Composite teeth	38.28	1.06	0.899	-54.10	130.65
	Acrylic teeth vs. CAD/CAM-milled teeth	-84.05	-2.32	0.084	-176.42	8.32
	Composite teeth vs. CAD/CAM-milled teeth	-122.33	-3.38	0.007*	-214.70	-29.96

The mixed ANOVA results indicated a statistically significant main effect for the group factor (denture base material) with a large effect size (η² = 0.25). This signifies that the choice between the conventional heat-cured denture base material and CAD/CAM-milled denture base material alone led to a significant difference in the overall mean shear bond strength. Furthermore, a highly significant interaction effect was found between the group and subgroup (tooth type), with a large effect size (η² = 0.28). This crucial finding means that the effect of tooth type on bond strength is not consistent but depends on the specific denture base material used. In contrast, the main effect for the subgroup was not statistically significant (p = 0.245), indicating that when averaged across both denture bases, the tooth materials alone did not produce significantly different bond strengths (Table 3).

**Table 3:** Two-way mixed analysis of variance (ANOVA) evaluating the effects of denture base material and artificial tooth type on shear bond strength. df: degree of freedom, *p < 0.05 denotes statistical significance.

Variables	Type III sum of squares	df	Mean square	F stats	p-value	Effect size
Denture base (Group)	101389.59	1	101389.59	17.69	0.001*	0.25
Tooth type (Subgroup)	16524.97	2	8262.48	1.44	0.245	0.05
Group x Subgroup	122477.49	2	61238.75	10.69	0.001*	0.28

The Bonferroni post-hoc test revealed specific pairwise differences across the six material combinations. The only statistically significant differences were found when comparing CAD/CAM-milled teeth bonded to a CAD/CAM-milled denture base, which demonstrated a significantly higher shear bond strength than acrylic teeth bonded to a conventional heat-cured denture base (p = 0.001) or CAD/CAM-milled teeth bonded to a conventional heat-cured denture base (p = 0.001). Furthermore, in the CAD/CAM-milled denture base group, CAD/CAM-milled teeth showed a significantly stronger bond than composite teeth (p = 0.01). No other pairwise comparisons were statistically significant after adjustment. This inference confirms that the interaction effect is driven by the superior performance of the matched CAD/CAM tooth-base combination. Using CAD/CAM teeth with a CAD/CAM denture base creates a significantly stronger bond than most other pairings, particularly any combination involving a conventional heat-cured denture base (Table 4).

**Table 4:** Bonferroni-adjusted pairwise comparisons of shear bond strength across all six material combinations. PMMA: polymethyl methacrylate, CAD/CAM: computer-aided design/computer-aided manufacturing, MPa: megapascals p-value adjusted for comparison of six groups, *p < 0.05 denotes statistical significance. Positive mean difference indicates the first combination has higher bond strength.

Pairwise comparison of groups and subgroups		Mean difference (MPa)	T stats	p-value
Acrylic teeth vs. Conventional heat-cured PMMA	Composite teeth vs. Conventional heat-cured PMMA	-93.02	-2.75	0.122
Acrylic teeth vs. Conventional heat-cured PMMA	CAD/CAM-milled teeth vs. Conventional heat-cured PMMA	4.62	0.14	1.000
Acrylic teeth vs. Conventional heat-cured PMMA	Acrylic teeth vs. CAD/CAM-milled PMMA	-96.42	-2.85	0.093
Acrylic teeth vs. Conventional heat-cured PMMA	Composite teeth vs. CAD/CAM-milled PMMA	-58.15	-1.72	1.000
Acrylic teeth vs. Conventional heat-cured PMMA	CAD/CAM-milled teeth vs. CAD/CAM-milled PMMA	-180.47	-5.33	0.001*
Composite teeth vs. Conventional heat-cured PMMA	CAD/CAM-milled teeth vs. Conventional heat-cured PMMA	97.64	2.88	0.084
Composite teeth vs. Conventional heat-cured PMMA	Acrylic teeth vs. CAD/CAM-milled PMMA	-3.41	-0.10	1.000
Composite teeth vs. Conventional heat-cured PMMA	Composite teeth vs. CAD/CAM-milled PMMA	34.87	1.03	1.000
Composite teeth vs. Conventional heat-cured PMMA	CAD/CAM-milled teeth vs. CAD/CAM-milled PMMA	-87.46	-2.58	0.188
CAD/CAM-milled teeth vs. Conventional heat-cured PMMA	Acrylic teeth vs. CAD/CAM-milled PMMA	-101.04	-2.98	0.064
CAD/CAM-milled teeth vs. Conventional heat-cured PMMA	Composite teeth vs. CAD/CAM-milled PMMA	-62.77	-1.85	1.000
CAD/CAM-milled teeth vs. Conventional heat-cured PMMA	CAD/CAM-milled teeth vs. CAD/CAM-milled PMMA	-185.10	-5.47	0.001*
Acrylic teeth vs. CAD/CAM-milled PMMA	Composite teeth vs. CAD/CAM-milled PMMA	38.28	1.13	1.000
Acrylic teeth vs. CAD/CAM-milled PMMA	CAD/CAM-milled teeth vs. CAD/CAM-milled PMMA	-84.05	-2.48	0.243
Composite teeth vs. CAD/CAM-milled PMMA	CAD/CAM-milled teeth vs. CAD/CAM-milled PMMA	-122.33	-3.61	0.010*

## Discussion

The results of this study highlight the critical role of material compatibility in achieving reliable tooth-to-base adhesions in complete dentures. The markedly superior bond strength observed when both the denture base and artificial teeth are milled from pre-polymerized high-pressure PMMA discs confirms that the homogeneity of polymerization degree and chemical composition is a dominant factor in bond performance. The highest bond strength was observed when CAD/CAM-milled teeth were used with a CAD/CAM-milled denture base. This finding is consistent with multiple recent investigations that reported significantly higher bond strengths with matched CAD/CAM systems compared to conventional combinations [12,13]. The strong chemical union achieved in such systems results in a quasi-monolithic structure, dramatically reducing the risk of interfacial failure. CAD/CAM PMMA materials are produced under meticulously regulated conditions, thereby guaranteeing consistent polymerization and reduced residual monomer levels, which are postulated to significantly enhance bond strength [14].

By contrast, when CAD/CAM-milled teeth were bonded to conventional heat-cured PMMA, the bond strength was among the lowest recorded values. This poor performance can be attributed to the highly polymerized and chemically inert ridge-lap surface of milled teeth, which resists swelling and diffusion of the methyl methacrylate monomer from the freshly packed heat-cured dough. Interpenetrating polymer network formation is severely limited without aggressive mechanical retention or specialized priming, resulting in predominant mechanical interlocking, which is prone to early failure [15]. In contrast to our findings, Prpić et al. [16] reported no significant difference in bond strength when CAD/CAM-milled teeth were bonded to conventional heat-cured dentures or CAD/CAM-milled dentures. Similar findings were reported by Han et al. [17], who concluded that resin teeth bonded to a CAD/CAM denture base showed similar bond strength as resin teeth bonded to a conventional denture base.

Interestingly, the composite teeth outperformed both acrylic and milled teeth when bonded to heat-cured bases, likely because of the presence of silanated fillers and multifunctional methacrylates that enhance wetting and co-polymerization with the free monomer present during conventional packing and curing. Similar findings were reported in a previous study by Chittaranjan et al. [11]. In contrast, a previous study reported the highest bond strength of acrylic teeth with heat-cured dentures compared with composite teeth [18]. 

However, this advantage disappeared when composite teeth were paired with CAD/CAM bases, reinforcing that no single tooth material is universally superior and performance is context-dependent on the base resin system. Tabassum et al. [19] performed a systematic review and meta-analysis and reported that the pooled bond strength obtained from the five included studies was 0.74, indicating that the bond strength of composite teeth demonstrated superiority when utilizing a CAD/CAM denture base compared to a heat-cured denture base. In summary, the findings were not statistically significant and exhibited considerable heterogeneity.

The significant interaction effect between denture base material and tooth type underscores that material selection decisions must be system-specific rather than tooth-centric. Traditional recommendations favoring highly crosslinked acrylic teeth for heat-cured bases may no longer hold in digital workflows. Laboratories and clinicians adopting milled or additively manufactured bases from pre-polymerized PMMA should prioritize teeth milled from the same material family, and strictly adhere to validated bonding protocols to maximize longevity and minimize one of the most common modes of complete denture failure.

The adhesion strength between denture base resins and artificial dentition is significantly contingent on the selection of bonding agents. The impact of various bonding agents on the adhesive strength of denture teeth was critically assessed by Cleto et al. [20]. The findings of this investigation indicated that adhesive strength was influenced by the specific type of bonding agent employed. An investigation conducted by Han et al. [17] revealed that CAD/CAM denture base materials bonded to denture teeth with resin cement demonstrated shear bond strengths similar to those achieved through conventional methodologies.

In this study, prefabricated denture teeth affixed to CAD/CAM denture base resins utilizing a PMMA bonding agent exhibited superior bond strength compared to those attached to heat-cured denture base resins. Although hot water bath polymerization continues to be the conventional method, contemporary bonding agents for CAD/CAM materials have been effectively employed to attain robust bond strength. Mine et al. [21] determined that bonding agents incorporating methyl methacrylate significantly enhance the adhesion of CAD/CAM resins. Furthermore, they discovered that the application of a methyl methacrylate monomer as a bonding agent on the ridge lap surfaces of acrylic denture teeth prior to processing can significantly improve the bond strength of denture base resins. It has been proposed that PMMA-based bonding agents facilitate bond strength in denture teeth by establishing a reactive interface within pre-polymerized CAD/CAM resins [9].

The clinical implications of these findings are clear: in fully digital workflows utilizing high-pressure polymerized PMMA bases, the use of correspondingly milled teeth from the same material line, bonded according to manufacturer instructions, offers the most reliable and durable tooth attachment currently available, and should be considered the new standard of care. Modern nanofilled composite teeth provide the strongest and most predictable adhesion among conventional or hybrid techniques, where heat-cured PMMA remains the base material. Mixing CAD/CAM-milled teeth with conventional heat-cured bases without additional surface activation (such as sandblasting, silicon coating, or application of adhesive monomers) should be avoided because they yield unacceptably weak bonds and increase the likelihood of early debonding.

The limitations of the study include its in vitro design with testing performed under dry conditions at room temperature without thermocycling or prolonged water storage, which may overestimate long-term clinical performance. Only one commercial system was evaluated for each category; therefore, the results may not be fully extrapolated to all available CAD/CAM or conventional materials. While standardized, shear loading does not replicate the complex cyclic, oblique, and fatigue forces encountered intraorally. Additionally, the relatively high standard deviations observed across several subgroups indicate considerable variability in bond strength, which reduces the precision of the estimates and limits the generalizability of the results. Finally, although the specimen preparation followed relevant specifications, minor variations in the bonded surface area and adhesive application technique between operators could introduce subtle bias. These factors restrict external validity, and therefore, the clinical recommendations drawn from this in-vitro study should be interpreted cautiously and validated through broader, multi-system investigations. Future investigations should include artificial aging, fatigue testing, and clinical follow-up to confirm the translational relevance of these laboratory findings.

## Conclusions

This study confirms that the shear bond strength between artificial teeth and denture base resin is strongly determined by material compatibility rather than by tooth type alone. The strongest and most reliable adhesion was achieved when both the base and teeth were milled from the same pre-polymerized high-pressure PMMA. Modern composite teeth provide superior bonding in conventional heat-cured workflows. Clinicians adopting digital complete denture protocols should prioritize matched CAD/CAM tooth-base combinations to minimize the debonding risk and enhance long-term clinical success.
